# Evaluating the Meditation Practices and Barriers to Adopting Mindful Medicine Among Physicians

**DOI:** 10.1177/15598276251323850

**Published:** 2025-03-12

**Authors:** Tiffany Champagne-Langabeer, Chelsea G. Ratcliff, Christine Bakos-Block, Francine Vega, Marylou Cardenas-Turanzas, Aila Malik, Radha Korupolu

**Affiliations:** 1 Center for Behavioral Emergency and Addiction Research, McWilliams School of Biomedical Informatics, The University of Texas Health Sciences Center at Houston (UTHealth), Houston, TX, USA (TCL, CBB, FV, MCT); 2 Department of Psychology, 4038 Sam Houston State University, Huntsville, TX, USA (CGR); 3 Department of Physical Medicine and Rehabilitation, McGovern School of Medicine, UTHealth Houston, Houston, TX, USA (AM, RK); 4 Department of Physical Medicine and Rehabilitation, TIRR Memorial Hermann Hospital, Houston, TX, USA (RK)

**Keywords:** meditation, clinical practice, survey, burnout

## Abstract

**Background:** Chronic pain affects over 25% of U.S. adults and is a leading cause of disability. Mindfulness meditation (MM) is a nonpharmacologic approach to manage pain and improve well-being. Despite mounting evidence supporting its efficacy, MM remains underutilized in medical practice. Understanding physicians’ engagement with MM and the barriers they face can inform strategies for integration into clinical care. This study assessed physicians’ attitudes toward MM, including barriers to practice and their likelihood of recommending it to patients. **Methods:** A cross-sectional survey of U.S. physicians was conducted from April to July 2024. Participants provided information on demographics, health struggles, and meditation practices and completed the Determinants of Meditation Practice Inventory-Revised to evaluate barriers. **Results:** Of 171 respondents, 37.4% meditated weekly, primarily for stress relief. Regular meditators were significantly more likely to recommend MM to patients (90.6%) compared to past (75%) or non-meditators (46.8%; *P* < .0001). Common barriers included time constraints (50.9%) and prioritizing other tasks (51.5%). Non- and past meditators reported low perceived benefits and inadequate knowledge (*P* ≤ .0001). **Conclusion:** Physicians’ engagement with MM influences their likelihood of recommending it. Addressing barriers through education, training, and promoting brief practices could enhance MM adoption and integration into clinical care.

“Our research found stress to be the primary motivator for regular meditation.”

## Introduction

Chronic pain, or pain lasting longer than 3 months, is one of the leading causes of disability in adults in the United States.^1^ Over 25% of adults in the U.S. suffer from chronic pain, and it is a primary complaint in outpatient medical care.^1^ The prevalence of chronic pain combined with the negative consequences of opioid medications has led to increased interest in complementary alternative medicine and therapies, such as mindfulness meditation (MM).^2^ Meditation practices stem from early Eastern traditions that date back thousands of years. These practices focus on mind and body integration and are used to improve overall well-being. Some types of meditation involve focusing on a particular sensation, such as breathing or maintaining awareness of the present moment, thereby facilitating a shift in attention from anticipatory and actual pain sensations to a more balanced mental state.^3^ From a neurobiological perspective, regular meditation practice has been shown to alter brain activity in areas related to pain and emotion regulation, potentially decreasing the perception of pain and improving coping strategies.^4^

Mindfulness meditation (MM) has increased in popularity among the general public in recent years. A report by the CDC in 2022 indicated that around 16.9% of adults in the U.S. practice MM, and the rate was highest within more affluent families.^5^ The growing availability of meditation apps on mobile platforms further highlights the rising popularity of MM with the public. A recent study revealed that apps for MM made up 96% of content in the health-focused app category.^6^ Several systematic reviews published on this topic have provided evidence that has informed decision-makers, healthcare administrators, and medical professional associations, contributing to the consideration of MM as a potential treatment option for a variety of conditions, including chronic pain.^7-9^ The Agency for Healthcare Research and Quality (AHRQ) conducted a comparative effectiveness review in 2014, finding moderate strength of the evidence supporting the efficacy and safety of MM among patients with stress-related outcomes such as anxiety and depression compared with active controls in diverse adult clinical populations.^10^

In response to this growing evidence, multiple prominent academic institutions have recognized MM’s potential to improve patients’ health and practitioners’ wellness with few risks and have created centers fostering research, education, and practice of MM.^11-14^ In 2017, the American College of Physicians (ACP) released guidelines recommending the use of nonpharmacologic treatments for chronic low back pain, including mindfulness-based interventions, over narcotics.^15^ Despite the evidence that MM improves wellness, its’ increasing adoption by the general public, and the interest in advancing its benefits to the clinical practice by a large body of decision-makers and health experts, MM remains marginalized within the conventional medical practice.^16,17^

The relatively slow integration of MM within conventional medical practice may be partly due to patient, provider, and system-level barriers to implementing a regular practice.^18^ The most common barrier reported by patients is a lack of time to practice MM, unfamiliarity with the practice, and emotional discomfort.^19-21^ Patients’ practice of MM is also greatly influenced by their provider’s attitude toward integrating holistic approaches into conventional treatment.^22^ For example, physicians experiencing burnout who participated in an 8-week course in MM reported decreased emotional exhaustion, depression, and stress and increased confidence in the benefits of the practice.^23^ Thus, examining provider’s personal attitudes toward meditation can shed light on strategies that may be particularly effective in enhancing the integration of meditation practices among healthcare professionals.

In this study, we assessed physicians’ reported barriers to meditation based on their own perceptions of meditation. By highlighting these barriers, this study seeks to inform targeted strategies to encourage meditation practices among healthcare professionals. We also aimed to benchmark physicians’ adoption of MM and their propensity to recommend MM to their patients.

## Methods

Participants were invited to participate in a confidential online survey about their meditation practices and views on recommending meditation to patients. We employed a snowball sampling approach, distributing the survey via institutional emails and closed professional groups on social media platforms such as Facebook and WhatsApp. Survey invitations were sent to physicians across multiple specialties and institutions, both academic and non-academic, to maximize diversity. Participants were encouraged to share the survey within their networks to broaden outreach. No demographic quotas were set during recruitment; however, we aimed to increase participation across specialties, geographic regions, and demographic characteristics by targeting a broad range of professional groups.

Inclusion criteria included physicians providing care to adults in the U.S. with a current medical license. Individuals provided informed consent electronically before completing the survey. Subsequently, participants were asked to answer an eligibility questionnaire, and only those who met eligibility were allowed to complete the remaining survey sections. Responses were collected from April 2024 to July 2024. Surveys were approved by the university’s Institution Review Board and Committee for the Protection of Human Subjects.

### Study Design

This anonymous cross-sectional survey was designed and delivered through REDCap™ (Research Electronic Data Capture), a web-based application, and took approximately less than 10 minutes to complete.^24^ Participants did not receive any compensation for participation.

### Survey Questionnaire

#### Demographic and Personal Health Information

Participants were asked to provide their age, gender, race, ethnicity, relationship status, work location, work setting, years of practice, and specialty certifications. We also asked the participants to indicate (yes/no) if they struggled with the following health issues: pain, headache, depression, anxiety, insomnia, stress, burnout, fatigue, other conditions (with a free-text option), or none of the above conditions.

#### Personal Meditation Practice

We asked the participants to indicate if they meditated at least once a week (yes/no). If participants responded “no,” they were asked if they had meditated at least once a week in the past or had never meditated. For those who meditated at least once a week or had done so in the past, we asked for details about the mode and methods of learning, reasons for meditating, duration, frequency, and how helpful the meditation practice was to them (response options provided in Table 2). We gathered the reasons for stopping meditation for those who previously meditated but abandoned this practice. All participants, regardless of their prior experience with meditation, were asked to indicate their interest in learning more about meditation and their preferred practice method. The options included (1) app-guided meditation, (2) virtual individual sessions, (3) virtual group sessions, (4) in-person individual sessions, and (5) in-person group sessions. An additional “other” option allowed participants to specify alternative preferences in an open text box, ensuring the survey captured a broad range of potential meditation formats.

**Table 1.** Sociodemographic Characteristics of Physicians Surveyed About Mindfulness Meditation Knowledge and Practices (*N* = 171).

Characteristic	*N* = 171 (100%)
Age, mean (sd) (*n* = 166)	44.78 (9.1)
Gender (*n* = 170)	
Female	106 (62.4)
Male	64 (37.6)
Race^a^ (*n* = 168)	
White	84 (50.0)
Black/African American	6 (3.6)
Asian	76 (45.2)
More than 1 race	2 (1.2)
Ethnicity (*n* = 168)	
Non-Hispanic/Non-Latino	150 (89.3)
Hispanic/Latino	18 (10.7)
Relationship status (*n* = 170)	
Married	133 (78.2)
Never married	15 (8.8)
A member of an unmarried couple	11 (6.5)
Divorced	9 (5.3)
Widowed	1 (.6)
Separated	1 (.6)
The region where currently practices medicine (*n* = 169)	
West	27 (16.0)
Midwest	14 (8.3)
Northeast	9 (5.3)
Southwest	87 (51.5)
Southeast	31 (18.3)
Puerto Rico^b^	1 (.6)
Work setting^c^ (*n* = 169)	
Academic	121 (71.6)
Private practice	37 (21.9)
Other	20 (11.8)
Years of medical practice (*n* = 170)	
0-5 years	46 (27.1)
6-10 years	37 (21.8)
11-15 years	31 (18.2)
>15 years	56 (32.9)
Specialty certifications^c^ (*n* = 170)	
Anesthesiology	4 (2.4)
Physical medicine and rehabilitation	107 (62.9)
Family medicine	4 (2.4)
Internal medicine/primary care	24 (14.1)
Pain medicine	4 (2.4)
Neurology	10 (5.9)
Psychiatry	3 (1.8)
General surgery	1 (.6)
Other	39 (22.9)
Health struggles (*n* = 171)^c^	
Pain	27 (15.8)
Headache	35 (20.5)
Depression	12 (7.0)
Anxiety	37 (21.6)
Insomnia	34 (19.9)
Stress	90 (52.6)
Burn out	68 (39.8)
Fatigue	68 (39.8)
Other	2 (1.2)
None	31 (18.1)
Prefer not to answer	8 (4.7)
Sum of health struggles (*n* = 132)^d^	
1-2	101 (76.5)
3-4	51 (38.6)
>4	19 (14.4)
Do you meditate at least once a week? (*n* = 171)	
Yes	64 (37.4)
No	107 (62.6)

Note: Numbers in rows do not add up to 171 due to missing values. Percentages in rows do not add up to 100 due to rounding.

^a^No American Indian/Alaskan Native or Native Hawaiian/other Pacific Islander participants responded to this survey.

^b^Puerto Rico was not included within the regions of the U.S., but we had one participant from that area.

^c^These categories are independent of each other, then the percentages do not add up to 100 because participants may have reported more than one category.

^d^Excluded participants reporting no health struggles and participants who refused to answer.

#### Recommendation of Mediation to Patients

Participants were asked if they recommended meditation to patients (yes/no) and the reasons for recommending or not recommending the practice (response options presented in Table 3).

**Table 2.** Survey Responses to Questions From Participants Who Meditated at Least Once a Week.

Current meditators	*N* (%)
How were you introduced to meditation? (*n* = 63)	
Learned through an in-person event	34 (54.0)
Learned on my own	29 (46.0)
What prompted you to start meditating?^a^ (*n* = 64)	
Feeling anxious/nervous	21 (32.8)
Frequent stress	35 (54.7)
Insomnia	6 (9.4)
Feeling depressed	2 (3.1)
Headache	1 (1.6)
Pain	1 (1.6)
Other	26 (40.6)
How helpful has meditation been for you? (*n* = 64)	
Very helpful	37 (57.8)
Helpful	26 (40.6)
Neither helpful nor unhelpful	1 (1.6)
What methods do you use?^a^ (*n* = 64)	
Self-guided	48 (75.0)
App-guided	33 (51.6)
Group session	14 (21.9)
Individual session	15 (23.4)
Other	5 (7.8)
How frequently do you meditate? (*n* = 64)	
1-2 times per week	30 (46.9)
3-4 times per week	10 (15.6)
>4 times per week	24 (37.5)
*Questions for participants who have never meditated*	
Are you interested in learning about meditation? (*n* = 59)	
Yes	37 (62.7)
No	22 (37.3)
If you were interested in meditation practices, what mode of delivery do you prefer? (*n* = 44)^a^	
App-guided	31 (70.5)
Self-guided	19 (43.2)
Virtual individual sessions	11 (25.0)
Virtual group sessions	7 (15.9)
In-person individual session	7 (15.9)
In-person group sessions	12 (27.3)

Note: Numbers in rows do not add up to 171 due to missing values. Percentages in rows do not add up to 100 due to rounding.

^a^These categories are independent of each other, then the percentages do not add up to 100 because participants may have reported more than 1 category.

#### Barriers to Practicing Meditation

Reliable and valid self-report instruments are available to evaluate barriers to MM in target populations. The Determinants of Meditation Practice Inventory (DMPI) was developed to assess meditation barriers.^25^ The instrument consists of 17 items representing barriers to MM and is coded as a five-point Likert scale where higher scores indicate agreement with the item. The DMPI has good psychometric properties and has been widely accepted by researchers. More recently, this instrument was revised and validated (DMPI-R) to add dimensionality to the DMPI by detecting types of barriers within the original inventory.^26^ The DMPI-R is a condensed version consisting of 12 of the original 17 questions from the DMPI and is also coded as a five-point Likert scale. Similar to the original DMPI, the validated DMPI-R has been widely used among adults from the U.S. and has allowed researchers to understand the needs of individuals, compare the barriers between groups, and evaluate the alterations in the prevalence of the barriers over time.

Participants completed the Determinants of Meditation Practice Inventory (DMPI), which was used to assess barriers to meditation.^25^ This inventory consists of 17 statements that complete the phrase “It will be difficult for me to meditate because…” Specific items were mapped to 4 components, each corresponding to a type of barrier, per the DMPI-R^26^: (1) Low Perceived Benefit, (2) Perceived Inadequate Knowledge, (3) Perceived Pragmatic Barriers, and 4)Perceived Sociocultural Conflict (Table 6 for items aligning with each of the 4 barriers).

**Table 3.** Reasons for Participants to Recommend or Not Recommend Meditation to Their Patients.

Question	*N* (%)
Have you recommended meditation to patients? (*n* = 170)	
Yes	120 (70.6)
No	50 (29.4)
If response was yes, reasons to recommend meditation^a^ (*n* = 119)	
Anxiety	94 (79.0)
General stress	82 (68.9)
Pain	75 (63.0)
General well-being	75 (63.0)
Sleep disorder	73 (61.3)
Depression	67 (56.3)
Post-traumatic stress disorder	50 (42.0)
Fatigue	42 (35.3)
Headache	34 (28.6)
Other	11 (9.2)
If response was no, reasons for not recommending meditation^a^ (*n* = 50)	
I don’t know much about meditation or mindfulness	28 (56.0)
I am not sure if my patients would be interested in this intervention	20 (40.0)
Lack of time to discuss non-pharmacological intervention	16 (32.0)
I don’t believe it will help my patients	1 (2.0)
Other reasons	9 (18.0)

^a^Responses are independent of each other; percentages do not add up to 100.

The DMPI-R exhibited the following reliability coefficients: Cronbach’s alpha for Low Perceived Benefit is .88; for Perceived Inadequate Knowledge, .84; for Perceived Pragmatic Barriers, .80; and for Perceived Sociocultural Conflict, .75. These coefficients indicate that the DMPI-R demonstrates acceptable to excellent internal consistency, supporting its reliability for assessing perceived barriers to meditation practice.^25,26^

### Statistical Analysis

Study participants were divided into 3 categories: current meditators (who meditate at least once a week), past meditators (who do not meditate currently but meditated at least once a week in the past), and non-meditators (those who never meditated). In describing the study’s variables, we reported frequencies and measures of central tendency for continuous variables; we reported frequencies and proportions for ordinal and nominal variables. We examined differences in demographic and personal health information, personal meditation practices, and recommendation of meditation to patients between current and past meditators. When comparing these groups, we conducted Fisher’s exact test for nominal or ordinal variables and Student’s T test for continuous variables if normally distributed. We also compared proportions of responses of “strongly agree” and “agree” to the barriers to meditation included in the DMPI among 3 groups: the current meditators, the past and the never meditators. When comparing these groups, we conducted Fisher’s exact test. Additionally, we examined differences in the mean scores of the 4 components of the DMPI-R (e.g., low perceived benefit, perceived inadequate knowledge, perceived pragmatic barriers, and perceived sociocultural conflict) between 2 groups: current meditators vs past and never meditators by conducting Student T test and reported the mean score difference (sd), and the 95% CI of the difference. When comparing groups, we determined familywise error rates with the Bonferroni correction adjusting the thresholds of the *P*-values and noting as significant the *P*-values under the respective adjusted threshold for significance. We also evaluated the effect size of the differences using Cohen’s d. Our cut points to determine the size of the effect were ≥.20 to <.50 (small), ≥.50 to <.80 (medium), and >.80 (large).^27^ A *P*-value <.05 (two-tailed when appropriate) was considered significant. All analyses were conducted using Stata I/C version 15, Stata Corp LLC, College Station, TX.

## Results

### Demographic and Personal Health Characteristics

Among the 186 eligible physicians who provided consent, 171 completed the survey. The mean (SD) age of the participants was 44.8 (9.1) years (*n* = 166). Ages ranged from 28 to 68 years. Most participants were 40-49 years old (*n* = 65, 39.1%). Of 170 participants, 62.4% (*n* = 106) were female. Half of the participants were White (*n* = 84, 50.0%), followed by Asian (*n* = 76, 45.2%), Black/African American (*n* = 6; 3.6%), and more than 1 race (*n* = 2; 1.2%). The majority worked in an academic setting (*n* = 121, 71.6%). Almost one-third of the participants had more than 15 years of medical practice experience (*n* = 56, 32.9%), and most specialized in physical medicine and rehabilitation (*n* = 107, 62.9%). Over half of the participants said they struggled with stress (*n* = 90, 52.6%), and one-third (*n* = 68, 39.8%) struggled with fatigue and burnout. Other challenges included anxiety (*n* = 37, 21.6%), headache (*n* = 35, 20.5%), insomnia (*n* = 34, 19.9%), and pain (*n* = 27, 15.8%). (Table 1).

**Table 4.** Demographic Characteristics and Meditation Practices of Participants Who Meditate Regularly and Participants Who Currently do Not Meditate but Meditated in the Past.

	Meditating regularly *N* (%)	Meditated in the past *N* (%)	Total	*P*-value
Total	64 (59.3)	44 (40.7)	108 (100)	
Demographic and professional characteristics				
Age, mean (sd)	45.4 (8.5)	44.8 (9.2)	45.1 (8.8)	.74
Gender				
Female	47 (73.4)	27 (61.4)	74 (68.5)	
Male	17 (26.6)	17 (38.6)	34 (31.5)	.21
Race				
Asian	35 (55.6)	19 (45.2)	54 (51.4)	
White	25 (39.7)	21 (50.0)	46 (43.8)	
Black/African American	2 (3.2)	2 (4.8)	4 (3.8)	
More than 1 race	1 (1.6)	0	1 (.9)	.60
Ethnicity				
Non-Hispanic/non-Latino	59 (92.2)	37 (88.1)	96 (90.6)	
Hispanic/Latino	5 (7.8)	5 (8.9)	10 (9.4)	.51
Work setting^a^	46 (71.9)	28 (63.6)21 (19.6)	74 (68.5)	.40
Academic private practice	16 (25.0)	2 (27.3)	28 (25.9)	.83
Other	7 (10.9)	5 (11.4)	12 (11.1)	.99
Years of medical practice				
0 – 5	16 (25.4)	12 (27.3)	28 (26.2)	
6 – 10	11 (17.5)	8 (18.2)	19 (17.8)	
11 – 15	14 (22.2)	7 (15.9)	21 (19.6)	
>15 years	22 (34.9)	17 (38.6)	39 (36.4)	.89
Meditation preferences and practice				
Have you recommended meditation to patients?				
Yes	58 (90.6)	33 (75.0)	91 (84.3)	
No	6 (9.4)	11 (25.0)	17 (15.7)	.03
How were you introduced to meditation?				
Learned through an in-person event^b^	34 (54.0)	25 (56.8)	59 (55.1)	
Learned on my own^c^	29 (46.0)	19 (43.2)	48 (44.9)	.84
What methods do you/did you use?^a^ Self-guided	48 (75.0)	24 (54.5)	72 (66.7)	.04
App-guided	33 (51.6)	20 (45.4)	53 (49.1)	.56
Group session	14 (21.9)	16 (36.4)	30 (27.8)	.13
Individual session	15 (23.4)	8 (18.2)	23 (21.3)	.63
Other	5 (7.8)	0	5 (4.6)	.08
Frequency of meditation				
1 – 2 per week	30 (46.9)	30 (69.8)	60 (56.1)	
3 – 4 per week	10 (15.6)	11 (25.6)	21 (19.6)	
>4 times per week	24 (37.5)	2 (4.6)	26 (24.3)	≤.001^d^
How helpful was meditation for you?				
Very helpful	37 (57.8)	5 (11.4)	42 (38.9)	
Helpful	26 (40.6)	30 (68.2)	56 (51.8)	
Neither helpful nor unhelpful	1 (1.6)	8 (18.2)	9 (8.3)	
Unhelpful	0	0	0	≤.001^d^
Very unhelpful	0	1 (2.3)	1 (.9)	

^a^Responses are independent of each other, percentages do not add up to 100.

^b^From a school, professional training, meditation group, or from a friend.

^c^Learned through an app, book, etc.

^d^Significant after calculating familywise error rate (Bonferroni correction).

Note: Demographic and professional characteristics adjusted *P*-value threshold ≤.006; Meditation preferences and practice characteristics adjusted *P*-value threshold ≤.005.

### Personal Meditation Practice

When physicians were asked if they meditated at least once a week, over a third said yes (37.4%, *n* = 64). Among current meditators, just over half indicated learning about meditation through in-person events (*n* = 34, 54.0%), and just under half on their own (*n* = 29, 46.0%). Current meditators indicated stress relief was the most prevalent reason for meditating regularly (*n* = 35, 54.7%), followed by anxious/nervous feelings (*n* = 21, 32.8%). Most current meditators found meditation helpful (*n* = 63, 98.4%), with self-guided (*n* = 48, 75.0%) as the learning method of choice (Table 2). A higher proportion of women were found among current meditators compared to those who had meditated in the past. Specifically, 73.4% of the current meditators were women (*n* = 47), compared to 61.4% (*n* = 27) in the past meditators group. After considering adjustment of *P*-values for familywise error rate, the frequency of meditation sessions per week and the perception of meditation being helpful were significant. A significantly higher proportion of current meditators had 4 or more sessions per week when compared to past meditators, 37.5% and 4.6%; respectively (*P*-value ≤.001). Regardless of meditation being helpful, the proportion of current meditators who consider meditation very helpful was 57.8% compared to only 11.4% of the past meditators (*P*-value ≤.001). No other significant differences were found among meditators and past meditators (Table 4).

**Table 5.** Responses of Physicians When Asked About Barriers to Meditation, Comparing Groups of Current, Past, and Never Meditators.

Barriers	Meditation group				
	Currently meditating *N* (%)^a^	Past meditator *N* (%)^a^	Never meditator *N* (%)^a^	Total *N* (%)^a^	*P*-value
Total	64 (37.4)	44 (25.7)	63 (36.8)	171 (100)	
Component: Low perceived benefit					
I prefer to accomplish something else	17 (26.6)	31 (70.4)	40 (63.5)	88 (51.5)	≤.001
Meditation might be boring	5 (7.8)	15 (34.1)	18 (28.6)	38 (22.2)	≤.01
It’s a waste of time to sit and do nothing	3 (4.7)	4 (9.1)	8 (12.7)	15 (8.8)	.28
I don’t believe meditation can help me	4 (6.2)	3 (6.8)	8 (12.7)	15 (8.8)	.46
Component: perceived inadequate knowledge					
I don’t know much about meditation	5 (7.8)	13 (29.5)	42 (66.7)	60 (35.1)	≤.001
I wouldn’t know if I am doing it right	5 (7.8)	12 (27.3)	26 (41.3)	43 (25.1)	≤.001
Component: perceived pragmatic barriers					
There is no quiet place where I can meditate	5 (7.8)	5 (11.4)	10 (15.9)	20 (11.7)	.35
I don’t have time	25 (39.1)	25 (56.8)	37 (58.7)	87 (50.9)	.06
There is never a time that I can be alone	7 (10.9)	13 (29.5)	15 (23.8)	35 (20.5)	.04
Component: perceived sociocultural conflict					
I am concerned meditation will conflict with my religion	1 (1.6)	1 (2.3)	1 (1.6)	3 (1.7)	.99
My family would think it was unusual	4 (6.2)	4 (9.1)	3 (4.8)	11 (6.4)	.67
I wonder if meditation might harm me	1 (1.6)	0	2 (3.2)	3 (1.7)	.63
Items recommended to clinicians to initiate discussions on the topic					
I am uncomfortable with silence	12 (18.7)	7 (15.9)	5 (7.9)	24 (14.0)	.20
I can’t stop my thoughts	23 (35.9)	22 (50.0)	25 (39.7)	70 (40.9)	.33
Additional questions asked for discussion					
I can’t sit still long enough to meditate	13 (20.3)	13 (29.5)	16 (25.4)	42 (24.6)	.54
Prayer is my form of meditation	18 (28.1)	11 (27.3)	12 (17.5)	41 (24.0)	.30
I would feel odd meditating	3 (4.7)	2 (4.5)	6 (9.5)	11 (6.4)	.53

^a^*N* and % represent the proportions who agreed and strongly agreed within responses to the full 5-point Likert scale; Significant after calculating familywise error rate (Bonferroni correction).

Note: Component: low perceived benefit adjusted *P*-value threshold ≤.004; Component: perceived inadequate knowledge adjusted *P*-value threshold ≤.008; Component: perceived pragmatic barriers adjusted *P*-value threshold ≤.005; Component: perceived sociocultural conflict adjusted *P*-value threshold ≤.005; Items recommended to clinicians to initiate discussions on the topic adjusted *P*-value threshold ≤.005; Additional questions asked for discussions adjusted *P*-value threshold ≤.005.

### Recommendation of Meditation to Patients

Most clinicians (*n* = 120, 70.6%) indicated that they had recommended meditation to patients. The most common reason for recommending the practice was patient anxiety (*n* = 94, 79.0%). Other reasons were general stress (*n* = 82, 68.9%), pain (*n* = 75, 63.0%), and general well-being (*n* = 75, 63.0%). Among those who indicated not having recommended meditation (*n* = 50), the leading reasons for not recommending meditation to patients included a lack of knowledge (*n* = 28, 56.0%), a lack of certainty surrounding patient interest in meditation or mindfulness practices, and a lack of time to discuss non-pharmacological interventions (*n* = 16, 32.0%). (Table 3).

When we compared current meditators (*n* = 64, 59.3%) with past meditators (*n* = 44, 40.7%), we found a higher proportion of physicians who were current meditators recommended meditation to patients (*n* = 58, 90.6%) as compared to physicians who were past meditators (*n* = 33, 75.0%. However, this comparison was not significant when adjusting the *P*-value threshold by using the familywise error rate (Table 4).

### Barriers to Meditation Practice

Participants’ responses to the barriers to the DMPI by groups of current meditators, past meditators and never meditators are presented in Table 5. After adjusting the *P*-values for familywise error rates, only 3 items showed significant differences. Approximately 1 quarter of current meditators indicated that “It will be difficult for me to meditate because…” they would instead accomplish something else (*n* = 17, 26.6%) compared to 70.4% and 63.5% of past and never meditators that indicated difficulty to meditate because of the same reason (*P*-value ≤.001). Not knowing much about meditation, and not knowing if they were doing it right were associated with significant differences among the groups after adjustment for familywise error rate. Lower proportions of current meditators indicated not knowing much about meditation (7.8%) compared to 29.5% of past meditators and 66.7% of participants who indicated they had never meditated (*P*-value ≤.001). Similarly, a lower proportion of 7.8% of current meditators indicated “I would not know if I am doing it right” compared 23.7% of past meditators and 41.3% of never meditators who indicated the same barrier (*P*-value ≤.001). Few participants felt that meditation would harm them (*n* = 3, 1.7%) or conflict with their religious beliefs (*n* = 3, 1.7%) (Table 5).

**Table 6.** Analysis of Barriers to Meditation by Inventory Components, Comparing Participants Who Currently Meditate Once a Week or More to a Combined Group of Those Who Meditated in the Past or Never Meditated.

Inventory component^26^	Meditation once a week *N* = 64	Meditation in the past or never *N* = 107	Score diff		*P*-value^a^ (adj. *P*-value)^b^	Size of effect	
	Mean (sd)	Mean (sd)	Mean (sd)	95% CI		Cohen’s d	95% CI
Component: low perceived benefit	1.80 (.72)	2.72 (.78)	.93	.69-1.16	≤.0001 (≤.0001)	1.22	.88-1.56 Large
Component: perceived inadequate knowledge	1.82 (.86)	3.03 (.90)	1.21	.93-1.49	≤.0001 (≤.0001)	1.36	1.02-1.70 Large
Component: perceived pragmatic barriers	2.13 (.81)	2.77 (.86)	.63	.37-.90	≤.0001 (≤.0001)	.75	.43-1.07 Medium
Component: perceived sociocultural conflict	1.32 (.58)	1.47 (.56)	.15	−.02-.33	.09 (.02)	.27	−.04-.58 Small

^a^Student T-test.

^b^Bonferroni correction controlling for familywise error rate; diff., difference; adj., adjusted; sd, standard deviation; CI, confidence interval.

Note: The adjusted *P*-value threshold calculated with the Bonferroni correction for the tests was ≤.006.

We averaged the items in each component to create a measure of perceived low benefit, inadequate knowledge, pragmatic barriers, and sociocultural conflict and compared endorsement of these 4 types of barriers for current meditators vs non-meditators (a combined group of past and non-meditators). After adjusting for familywise error rate, the groups significantly differed in their endorsement of 3 of the 4 components indicated (*P* ≤ .0001), with past and non-meditators indicating more robust agreement with each barrier category than current meditators. Specifically, the differences in endorsement of low perceived benefit and inadequate knowledge were associated with a large effect size (Cohen’s d = 1.22, 95% CI = .88-1.56) and (Cohen’d = 1.36, 95% CI = 1.02-1.70), respectively. In contrast, the difference in endorsement of perceived pragmatic barriers was associated with a medium effect size (Cohen’s d = .75, 95% CI = .43-1.07). No significant difference was observed between endorsement of perceived sociocultural conflict (Cohen’s d = .27, 95% CI = −.04-.58). Details are shown in Table 6.

## Discussion

This research assessed physician’s attitudes toward meditation, including their personal meditation practices, perceived barriers to meditation, and their practice of recommending mindful meditation to their patients. We found that just over one-third (*n* = 64, 37.4%) of those surveyed meditate regularly once per week. Those who meditated found it beneficial, and most used self or app-guided methods and reported meditating for over 10 minutes per session. Our research also found stress to be the primary motivator for regular meditation. In comparison, physicians who had never meditated expressed interest in learning (*n* = 37/59, 62.7%), indicating a potential willingness to explore meditation to improve overall health.

One of the most striking findings from our study was that although most physicians reported they had recommended meditation to their patients (70.6%, *n* = 120), those who had not recommended meditation identified several barriers, including a lack of knowledge about meditation, being unsure of patient interest in meditation, and lack of time to discuss meditation. Interestingly, only 1 participant indicated concern that meditation may not help their patients. These results indicate that more education is needed on MM’s flexibility and adaptability to individual contexts.

Given that physicians’ perceived obstacles to their personal MM practice influence their decision to recommend MM to their patients, 31 it is valuable to examine physicians’ perceptions of barriers to their meditation practice. In this study, physicians reported that the most common barriers to mediation included preferring to accomplish something else and not having time to meditate, which points to the limited time available to physicians. Approximately 40% of respondents indicated they could not stop their thoughts. The limited time available to engage in a stress-relieving practice may also be related to stress and burnout among physicians. Physicians experiencing burnout are less likely to engage in self-care practices such as mindfulness,^28^ suggests that physicians who may stand to derive the most significant benefit from meditation may be missing out due to high stress levels may extend to their willingness to encourage patients to try these methods. These common barriers suggest that greater education about the benefits of even brief meditation practices could be valuable for physicians. For example, 10-15 minutes of daily MM for 1 to 8 weeks is associated with improved mood and emotion regulation, and some studies suggest that even 5 minutes of daily MM for a week can have benefits.^29-31^ Additionally, among non-meditators, not knowing much about meditation and not knowing if they were doing it right were common barriers. This suggests that additional education in the form of experiential training (e.g., practicing meditation during training) would likely be particularly beneficial for novice physician meditators.

Continuing education about the benefits of meditation and mindfulness during medical training and residency may significantly improve physicians’ confidence in recommending it to their patients. Physicians are more likely to recommend interventions they understand and feel comfortable discussing with patients. A mindfulness-based intervention curriculum can enhance physicians’ knowledge and skills, making them more willing to recommend it.^32,33^ However, physicians may rely more on empirical evidence and clinical guidelines than personal practice in making recommendations. The landmark report *“To Err is Human: Building a Safer Health System”* (1999) by the Institute of Medicine (now the National Academy of Medicine) established clinical guidelines as the gold standard in promoting consistency in patient care and reducing medical errors.^34^ On balance, in a more recent Commentary published in the *AMA Journal of Ethics,* the authors contend that there are circumstances where a physician’s personal experience may eclipse guidelines provided there is patient-centered care, shared decision-making, and transparency of risks involved.^35^

There are limitations to this study, including potential selection bias due to the use of social media and email distribution for survey dissemination, which may have led to an overrepresentation of physicians active in online networks or professional organizations where mindfulness-related topics are commonly discussed. While we aimed to mitigate this by encouraging broad sharing across diverse institutions and specialties, future studies will incorporate additional recruitment methods, such as outreach through physician registries and professional societies, to ensure more comprehensive representation. Additionally, many of the physicians we surveyed worked in academic settings, specializing predominantly in Physical Medicine and Rehabilitation, and results may not be generalizable to the greater physician community. The type of medical setting may influence the integration of meditation into current medical practice. There may have been non-response or participation bias, and the participants in our survey may not reflect the views of those not responding. Future studies will aim to enhance sampling balance by utilizing physician registries, applying demographic quotas, and engaging with professional societies to ensure representation across age, gender, specialty, and practice settings.

## Conclusion

This research addresses gaps in the existing literature by exploring both the personal use of MM among physicians and their subsequent recommendations of this practice to their patients. While the bulk of research has focused on the general benefits of MM in healthcare, few have specifically examined how physicians’ own MM practices influence their professional behavior. By bridging the gap between personal practice and clinical application, this research provides a more comprehensive understanding of how mindfulness can be integrated into patient care.
